# Deletion of myosin VI causes slow retinal optic neuropathy and age-related macular degeneration (AMD)-relevant retinal phenotype

**DOI:** 10.1007/s00018-015-1913-3

**Published:** 2015-05-06

**Authors:** Timm Schubert, Corinna Gleiser, Peter Heiduschka, Christoph Franz, Kerstin Nagel-Wolfrum, Ayse Sahaboglu, Nicole Weisschuh, Gordon Eske, Karin Rohbock, Norman Rieger, François Paquet-Durand, Bernd Wissinger, Uwe Wolfrum, Bernhard Hirt, Wibke Singer, Lukas Rüttiger, Ulrike Zimmermann, Marlies Knipper

**Affiliations:** 1 Werner Reichardt Centre for Integrative Neuroscience (CIN)/Institute for Ophthalmic Research, University of Tübingen, Otfried-Müller-Str. 25, 72076 Tübingen, Germany; 2 Institute of Anatomy, Department of Clinical Anatomy and Cell Analysis, University of Tübingen, Elfriede-Aulhorn-Str. 8, 72076 Tübingen, Germany; 3 Experimental Vitreoretinal Surgery, Institute for Ophthalmic Research, Centre for Ophthalmology, University of Tübingen, 72076 Tübingen, Germany; 4 Present Address: University Eye Hospital Münster, Westfälische Wilhelms-University of Münster, Albert-Schweitzer-Campus 1, 48149 Münster, Germany; 5 Molecular Physiology of Hearing, Tübingen Hearing Research Centre, Department of Otolaryngology, Head and Neck Surgery, University of Tübingen, Elfriede-Aulhorn-Str. 5, 72076 Tübingen, Germany; 6 Institute of Zoology, Cell and Matrix Biology, Johannes Gutenberg University of Mainz, Johann-Joachim-Becher-Weg 11, 55128 Mainz, Germany; 7 Cell Death Mechanisms Group, Centre for Ophthalmology, Institute for Ophthalmic Research, University of Tübingen, Röntgenweg 11, 72076 Tübingen, Germany; 8 Molecular Genetics, Centre for Ophthalmology, Institute for Ophthalmic Research, University of Tübingen, Röntgenweg 11, 72076 Tübingen, Germany

**Keywords:** Bipolar cell, Inner retina, Outer retina, Synapse, Stereocilia, Translocator protein TSPO, Choriocapillaris, Mouse model

## Abstract

The unconventional myosin VI, a member of the actin-based motor protein family of myosins, is expressed in the retina. Its deletion was previously shown to reduce amplitudes of the a- and b-waves of the electroretinogram. Analyzing wild-type and myosin VI-deficient Snell’s Waltzer mice in more detail, the expression pattern of myosin VI in retinal pigment epithelium, outer limiting membrane, and outer plexiform layer could be linked with differential progressing ocular deficits. These encompassed reduced a-waves and b-waves and disturbed oscillatory potentials in the electroretinogram, photoreceptor cell death, retinal microglia infiltration, and formation of basal laminar deposits. A phenotype comprising features of glaucoma (neurodegeneration) and age-related macular degeneration could thus be uncovered that suggests dysfunction of myosin VI and its variable cargo adaptor proteins for membrane sorting and autophagy, as possible candidate mediators for both disease forms.

**Electronic supplementary material:**

The online version of this article (doi:10.1007/s00018-015-1913-3) contains supplementary material, which is available to authorized users.

## Introduction

The unconventional myosin VI, a member of the actin-based myosin motor protein family [1], is a reverse-directed myosin motor that moves towards the minus end of actin filaments [2]. Myosin VI is expressed in many tissues and cell types including hair cells of the cochlea [3] and retinal photoreceptor cells and the retinal pigment epithelium [4, 5]. In hair cells of the cochlea, myosin VI displays dual functions: (1) targeting of protein cargoes to stereocilia, the disturbance of which leads to deafness [6], (2) targeting of cargoes that couple exocytosis and endocytosis, the disturbance of which leads to cell surface shrinkage and disturbed replenishment of vesicles [7, 8]. In the central nervous system, previous studies suggested a role of myosin VI for coupling the membrane cycle towards cellular degrading processes (autophagy) and functional relevant membrane sorting through coupling to different cargo adaptor proteins [9]. In the retina of myosin VI-deficient Snell’s Waltzer mice, a-wave and b-wave amplitudes of the electroretinogram (ERG) were found to be reduced, although the overall retinal morphology and photoreceptor ultra-structure appeared normal [5]. However, the details of the pathology behind the reduced amplitudes of the a-wave and b-wave of the ERG described in this myosin VI mutant [5] and another mutant carrying a missense mutation in the motor domain of myosin VI [10] are elusive.

To study the role of myosin VI in the retina, we here performed scotopic and photopic ERG measurements in combination with high-resolution fluorescence and electron microscopy studies in young and aged myosin VI mutant mice. A subtle but nonetheless clear ocular phenotype with characteristics that resembles late onset optic neuropathy as well as age-related macular degeneration (AMD) has been observed. Deficits in myosin VI mutants included subretinal pigment epithelia basal laminar deposits [11], photoreceptor cell death, and microglial infiltration in the outer and inner retina [12–14]. Thus, this study suggests that disturbance of the myosin VI-mediated membrane cycle process may be a potential risk factor for the generation of AMD, glaucoma, or the described co-morbidity of both [15].

## Materials and methods

### Animals

Homozygous (sv/sv, myosin VI^sv/sv^) and heterozygous (+/sv, myosin VI^+/sv^) Snell’s Waltzer myosin VI-deficient mutant mice [3, 16] on a C57BL/6J background and appropriate littermate controls (wild-type) [5, 16] of both genders at ages of 1–1.5, 5–8, and more than 12 months were analyzed functionally and morphologically.

The care and use of animals were approved by the University of Tübingen, Veterinary Care Unit and the Animal Care and Ethics Committee of the regional board of the Federal State Government of Baden-Württemberg, Germany, and followed the guidelines of the EU Directive 2010/63/EU for animal experiments.

### Genotyping of the *rd8* mutation

Genomic DNA was isolated from mouse external ear biopsies using the QIAamp DNA Mini Kit (Qiagen). The *rd8* mutant and wild-type allele were distinguished after PCR amplification (5′-GCCCCTGTTTGCATGGAGGAAACTTGGAAGACAGCTACAGTTCATAT-3′, 5′-GCCCCATTTGCACACTGATGAC-3′) by the presence of a *Nde*I restriction site, present only in the *rd8* mutant allele. Digestion of the 244 bp PCR product with *Nde*I yields two characteristic fragments of 199 and 45 bp.

### Electrophysiology

ERG measurements were performed in 1-, 5–7-, and 12-month-old wild-type, and +/sv and sv/sv myosin VI mutant mice (*n* = 10 for each group). Animals were dark adapted for at least 24 h. They were anesthetized by an intraperitoneal injection of a mixture of ketamine and xylazine (120 mg/kg ketamine, 10 mg/kg xylazine). The cornea of the eyes of anesthetized mice was desensitized with a drop of Novesine (Novartis Ophthalmics). The upper eyelids were retracted slightly by a surgical silk thread. The animals were placed onto a heated platform (37 °C) during the measurements to keep their body temperature constant. Gold wire ring electrodes placed onto the corneas of both eyes served as working electrodes. A gold wire ring electrode was placed in the mouth to serve as a reference electrode. A stainless steel needle electrode was inserted into the tail of the animals for grounding. The pupils were dilated with a drop of Tropicamide (Novartis Ophthalmics). All these manipulations were performed under dim red light, without bringing the animals into ambient light after dark adaptation. The red light was switched off after finishing all the stages of animal preparation. After an additional 5 min to allow the pupils to dilate, measurement was started using the commercial RetiPort32 device from Roland Consult Systems (Brandenburg, Germany). Standard ERG measurements were performed, with scotopic flash ERG at up to eight different light intensities from 0.0003 to 100 cd s/m^2^, an additional run for scotopic oscillatory potentials at 100 cd s/m^2^, photopic 30 Hz Flicker at 3 cd s/m^2^ after 10 min of light adaptation, a photopic flash ERG, and photopic oscillatory potentials. The light intensity used for the flashes in the photopic ERG measurements was 100 cd s/m^2^. Data were collected over 160 ms per measurement, with 512 data points per measured waveform. The analog filters of the ERG device were set to the frequency ranges of 0.5–200 Hz for both scotopic and photopic flash ERG, 50–500 Hz for oscillatory potentials and 10–50 Hz for 30 Hz Flicker. In addition, the waveforms of the oscillatory potentials were digitally filtered offline using a DSP filter included in the software of the ERG device (−15 dB for *f* < 10 Hz). Amplitudes of a-waves were measured from the baseline to the bottom of the a-wave trough, whereas b-wave amplitudes were measured from the bottom of the a-wave trough to the peak of the b-wave. ERG measurements were performed simultaneously on both eyes in each animal, and the eye giving the better parameters was chosen for data evaluation. Values of amplitudes and implicit times of oscillatory potentials are given as so-called oscillatory indices, i.e., values of amplitudes and implicit times of the first four oscillations were summed up. Statistical significance of differences between the values obtained in the three experimental groups was checked by the Mann–Whitney test using the Prism 6 program by GraphPad Software, Inc. Data are presented as Tukey box plots.

### Histology and cell death labeling

For the analysis of cell death markers, wild-type, and +/sv and sv/sv mutant mice were used at 1.5 and 5–6 months of age. Eyes were enucleated, fixed with 4 % paraformaldehyde, cryoprotected in graded sucrose solutions, and embedded with Jung tissue freezing medium (Leica Microsystems, Wetzlar, Germany). The frozen eyes were sectioned (12 µm) in an HM560 cryotome (Microm, Walldorf, Germany). Detection of dying cells was performed as reported previously [17] using the terminal deoxynucleotidyltransferase (TdT) dUTP nick end labeling (TUNEL) assay (Roche Diagnostics, Mannheim, Germany). Microscopy was performed on a Zeiss Imager Z1 Apotome Microscope; images were captured with Zeiss Axiovision 4.7 software (Zeiss, Wetzlar, Germany).

For quantification of cell death, pictures were taken from three entire sagittal sections for at least three different animals for each genotype using the Mosaic mode of Axiovision 4.7 at 20× magnification. The average area occupied by a photoreceptor cell (i.e., soma size) for each genotype was determined by counting DAPI-stained nuclei in 9 different areas (50 × 50 µm) of the retina. The total number of photoreceptor cells was estimated by dividing the outer nuclear layer area by this average cell size. The number of positively labeled cells in the outer nuclear layer was counted manually.

Data were evaluated using Graph Pad Prism software (GraphPad Software, La Jolla, CA, USA) and ANOVA analysis with a Kruskal–Wallis test and Dunn’s multiple comparison post-test. Data are presented as a mean ± standard error of the mean (SEM).

### Immunohistochemistry

Retinae from adult (5–8- and 12–24-month-old) wild-type, and +/sv and sv/sv mutant mice for immunolabeling experiments were used. Retinae prepared for cryosections were dissected and fixed for 2 h with 2 % paraformaldehyde, dehydrated in increasing concentrations of sucrose, and then embedded in Tissue-Tek, and cryosectioned at a thickness of 12 µm. Sections were embedded with Vectashield mounting medium with DAPI (Vector Laboratories). Antibodies against myosin VI (rabbit, Santa Cruz, diluted 1:200), calbindin (mouse, Sigma-Aldrich, diluted 1:100), VGLUT1 (guinea pig, Synaptic Systems, diluted 1:100), PKCα (mouse, Novus Biologicals, diluted 1:100), AP-2 (mouse, BD Transduction, diluted 1:100), IBA-1 (rabbit, Wako, diluted 1:100), and translocator protein TSPO, also known as peripheral benzodiazepine receptor PBR (rabbit, abcam, diluted 1:100) were used. Primary antibodies were detected with Cy3-conjugated (Jackson ImmunoResearch Laboratories) or Alexa Fluor 488-conjugated secondary antibodies (Molecular Probes). Immunohostochemistry was repeated at least twice. Sections were viewed using an Olympus BX61. Images were acquired using a CCD camera and analyzed with cellSens Dimension software (OSIS GmbH, Münster, Germany). To increase spatial resolution, retinal sections were imaged over a distance of 8 μm within the outer and inner plexiform layer in an image-stack along the *z*-axis (*z*-stack). Typically, *z*-stacks consisted of 30 layers with a *z*-increment of 0.276 μm, for each layer one image per fluorochrome was acquired. *z*-stacks were 3-dimensionally deconvoluted using Cell^∧^F’s RIDE module with the Nearest Neighbour algorithm (OSIS). Figures show composite images, which represent the maximum intensity projection over all layers of the *z*-stack. Images were processed with Photoshop CS (Adobe Systems). Subcellular localization of immunohistochemically labeled myosin VI was visualized with a Zeiss 510 Meta confocal laser scanning microscope (Zeiss, Göttingen/Jena, Germany).

For quantification of immunopositive-IBA-1 microglia, immunohistochemistry was performed on retinal sections of 6–8-month-old (*n* = 3 animals) and 12–18-month-old (*n* = 3 animals) wild-type mice and 6–8-month-old (*n* = 4 animals) and 15–24-month-old (*n* = 4 animals) sv/sv myosin VI mutant mice. IBA-1 immunopositive cells were counted in the outer nuclear layer (ONL) and ganglion cell layer (GCL) using an integrated microscopic counting chamber to fix the region of interest (ROI). 15 different ROIs were analyzed for each retinal section. The number of IBA-1 positive cells per 100 µm retinal length was averaged for the ONL and RGC for each retinal section. The counting procedure was done 2–4 times for each animal. Finally, the average of IBA-1 immunopositive microglia cells for the ONL and the RGC per animal was calculated. Statistical analysis was performed using the 2-way ANOVA test with *α* = 0.05, followed by post hoc tests with Bonferroni adjustment for genotype and age. Data are presented as mean ± standard deviation and number of animals.

### Transmission electron microscopy (TEM)

Ultrastructural analysis of murine photoreceptor cells were performed as previously described [18]. Ultrathin sections were counterstained with ethanolic uranyl acetate and lead citrate, and analyzed in transmission electron microscopes (Tecnai 12 BioTwin, FEI, The Netherlands; LEO EM912 Omega electron microscope, Zeiss, Göttingen, Germany). Images were obtained with a CCD camera (SIS MegaView3; Surface Imaging Systems, Herzogenrath, Germany) and a slow-scan CCD camera (PROSCAN, Germany; analySIS pro imaging software, version 3.2) and processed with Adobe Photoshop CS (Adobe Systems).

## Results

### Retinal function is affected in myosin VI mutant mice

In previous studies, it has been reported that the absence of myosin VI leads to reduced a-wave and b-wave amplitudes of the ERG response in myosin VI-deficient sv/sv mice aged approximately 1.5 and 8.5 months [5]. Pathologic changes in outer retinal function can be detected individually for rod and cones upon differentiating scotopic and photopic Ganzfeld ERG [19]. In the present study, measurements performed in 1-month-old sv/sv mice were inconspicuously displaying normal ERG waves. However, in 5–6-month-old sv/sv mice, we found a significant reduction of a-wave and b-wave amplitudes for both scotopic and photopic conditions (data not shown) implying an effect on rod and cone photoreceptors observed in sv/sv mice within the first 6-month life span.

### Genotyping of the *rd8* mutation in the myosin VI mutant mice

Recently, it has been reported that the *rd8* mutation in the *Crb1* gene may confound ocular-induced mutant phenotypes, being responsible for multiple previously described retinal dystrophy or photoreceptor degeneration mouse models, instead of the predicted gene of interest [20, 21]. To rule out the possibility that the observed ocular phenotype in the myosin VI mutant mouse was due to the *rd8* mutation, DNA samples from three wild-type control mice, three +/sv and three sv/sv mutant mice were analyzed for the *rd8* mutation by PCR. As a positive control, we used DNA sampled from the Ccl2/Cx3cr1 double knockout (CCDKO) mouse line which is homozygous for the *rd8* mutation. The *rd8* allele was absent in all tested experimental animals (Fig. S1). Thus, an involvement of the *rd8* mutation in the ocular phenotype in myosin VI mutant mice can be ruled out.

### Myosin VI immunoreactivity is found in the pigment epithelium, the outer limiting membrane and outer plexiform layer

**Fig. 1:**
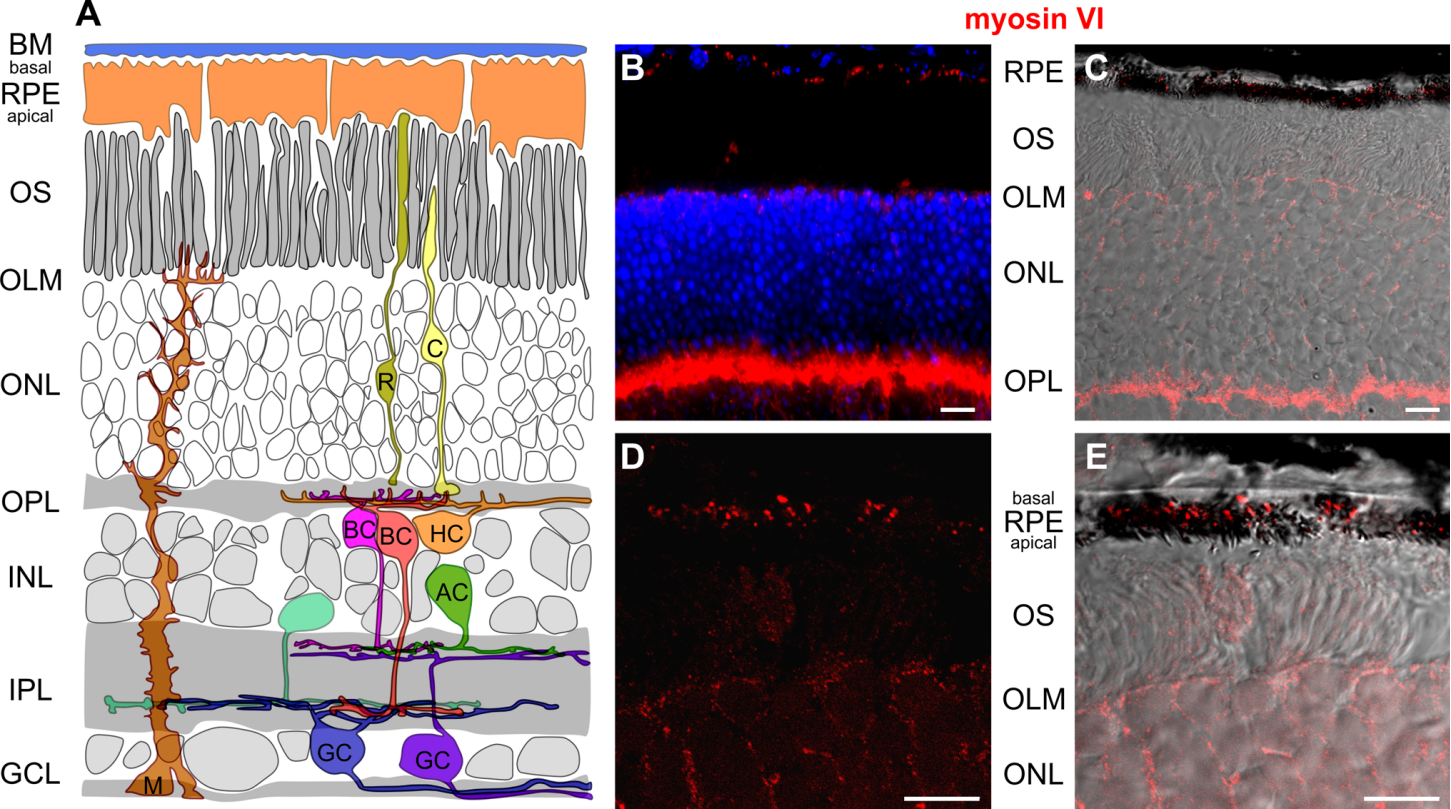
Immunolabeling of myosin VI in wild-type retina. **a** Scheme of the retina showing retinal layers and cell types. *BM* Bruch’s membrane, *RPE* retinal pigment epithelium, *OS* photoreceptor outer segment, *OLM* outer limiting membrane, *ONL* outer nuclear layer, *OPL* outer plexiform layer, *INL* inner nuclear layer, *IPL* inner plexiform layer, *GCL* ganglion cell layer, *C* cone, *R* rod, *HC* horizontal cell, *AC* amacrine cell, *M* Müller cell, *BC* bipolar cell, *GC* ganglion cell. **b** Immunolabeling of myosin VI (*red*) in wild-type retina shows myosin VI immunoreactivity in the RPE, OLM, ONL, and OPL. *Blue* DAPI nuclear labeling. **c** Merged images of myosin VI (*red*) immunolabeling and the corresponding differential interference contrast (DIC) image in wild-type retina showing the myosin VI labeling in the RPE cells, OLM, ONL, and OPL. **d** Confocal microscopic image (cLSM) of the subcellular localization of myosin VI in the RPE. **e** Merged image of the myosin VI immunolabeling in (**d**) and DIC. The high-magnification cLSM images show myosin VI immunoreactivity in the basal compartment of RPE cells. The myosin VI immunofluorescence signal in the OLM is out of the focal plane. The shown retinal myosin VI expression pattern was found in 7 different wild-type animals. *Scale bars* 10 µm

**Fig. 2:**
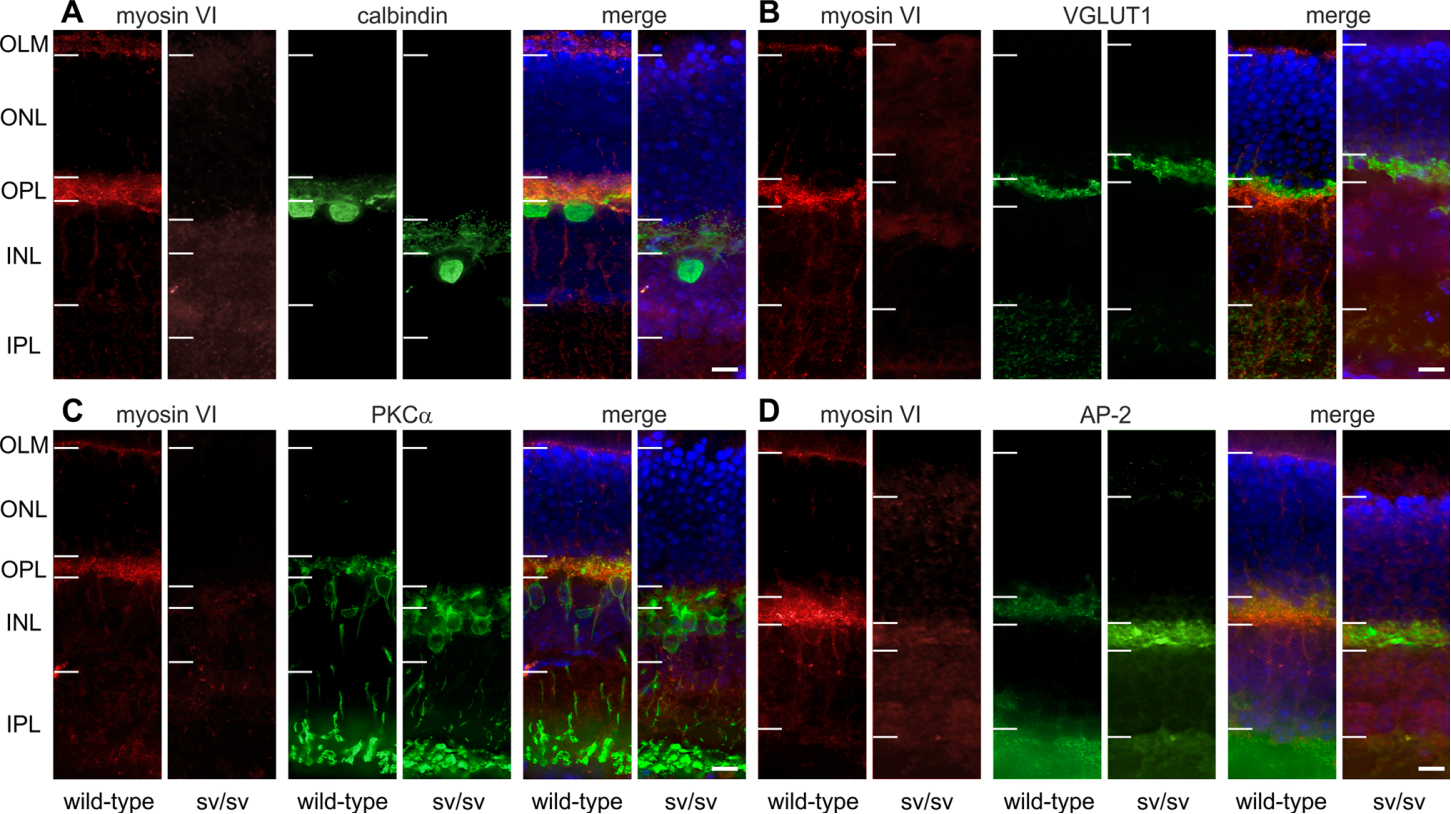
Immunohistochemistry in wild-type and sv/sv retinae of 5–6-month-old animals. **a**–**d** In wild-type mice, myosin VI immunoreactivity is found in the outer limiting membrane (OLM), outer plexiform layer (OPL), and to a weaker extent in the ONL. Immunoreactivity is absent in sv/sv mutant mice. Co-immunostainings were performed for **a** calbindin, a marker for horizontal cells, **b** VGLUT1, a marker for glutamatergic photoreceptor axon terminals, **c** PKCα (protein kinase C α), a marker for rod bipolar cells, and **d** AP-2 assembles with myosin VI. *Horizontal bars* indicate nuclear and synaptic layer boundaries. Due to the different locations of the vertical sections in the retina, the thickness of the layers appears to be distinct. However, no systematic difference between wild-type and sv/sv retinae was observed. *Scale bars* 10 µm

In 5–6-month-old wild-type mice, myosin VI protein immunoreactivity was localized in the retinal pigment epithelium (RPE), at the level of the outer limiting membrane (OLM) and the photoreceptor inner segments, the outer nuclear layer (ONL), and at the level of photoreceptor axon terminals in the outer plexiform layer (OPL) (Figs. 1b, c, 2, 7). Since RPE cells are a complex polar structure with apical microvilli contacting photoreceptor outer segments and basal infoldings contacting the Bruch’s membrane and the choroid [22], we analyzed the subcellular localization of myosin VI in RPE cells and found that myosin VI is mostly expressed at the level of the basal infoldings (BI) indicating expression predominantly in the basal compartment of RPE cells (Fig. 1d, e). At the level of the outer plexiform layer (OPL), a co-stratification of myosin VI was found with the calcium-binding protein calbindin (Fig. 2a), a marker for mouse horizontal cells and their processes contacting photoreceptor axon terminals [23]. Within the OPL, myosin VI immunoreactivity was also found at the level of the vesicular glutamate transporter 1 (VGLUT1) (Fig. 2b), a specific marker for glutamatergic photoreceptor terminals [24]. Additionally, myosin VI was partly co-localized with protein kinase C ∝ (PKCα) immunoreactivity (Fig. 2c), a specific marker for rod bipolar cells and their dendrites contacting rod terminals [23]. These findings provide undescribed hints for an expression of myosin VI in presynaptic and/or postsynaptic neurite elements of cells stratifying in the OPL. A co-localization of myosin VI within this region with the adaptor protein 2 (AP-2) (Fig. 2d), known to be involved in clathrin-mediated endocytosis through assembly with myosin VI [25], supports this observation. In contrast, myosin VI immunoreactivity in sv/sv mutant mice of the same age was absent (Fig. 2a–d). Taken together, myosin VI immunoreactivity is found in the RPE, the OLM, the ONL, and the OPL.

### Photoreceptors degenerate in myosin VI mutant mice

Photoreceptor loss is a characteristic feature of blinding diseases, including hereditary forms such as retinitis pigmentosa [26], but also in AMD [27–29]. To test whether the observed changes of retinal function [5] correlated with a degenerative phenotype, TUNEL staining was performed on retinae from 1.5- to 5–6-month-old +/sv and sv/sv mutant mice with wild-type littermates as control. Although there was a trend at the age of 1.5 months (Fig. S2A, C), we observed a small, but significant increase of cell death of photoreceptors in the outer nuclear layer (ONL) in 5–6-month-old sv/sv mutants (Fig. S2B, D). This finding suggests that loss of myosin VI triggered a slow progressive photoreceptor neurodegeneration.

### Myosin VI deletion leads to basal laminar deposits in the sub-retinal pigment epithelium

**Fig. 3:**
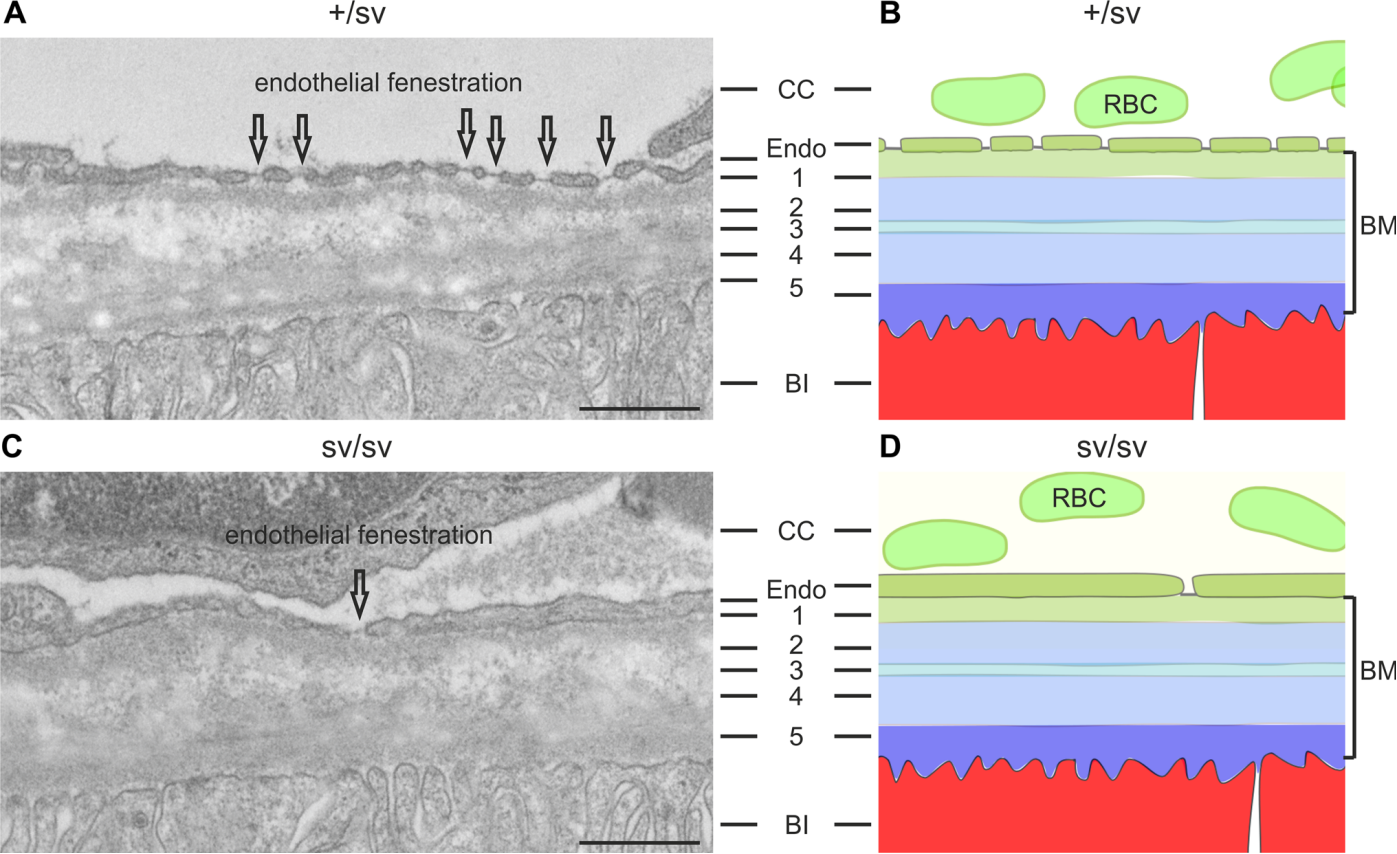
Transmission electron microscopy of outer +/sv and sv/sv retinae retinae of 5–6-month-old mice. **a** The RPE basal infoldings (BI), the pentalaminar-organized Bruch’s membrane (BM): *1* basal lamina of the choriocapillary (CC), *2* outer collagenous layer, *3* elastic layer, *4* inner collagenous layer, *5* basal lamina of the RPE, and the regular endothelial fenestration (indicated by *arrows*) of the CC are clearly visible in the +/sv retina. **b** Schematic illustration of (**a**). **c** In sv/sv mice, RPE BI are present; however, reduced fenestration of the CC and an increasing distance between remaining fenestrae was noted (*arrow*). **d** Schematic illustration of the morphological alterations in sv/sv mutant retina. *RBC* red blood cells, *Endo* endothelial cells. *Scale bars* 0.5 µm

At 5–6-months of age, the outer retina of heterozygous +/sv mice (Fig. 3a) showed no morphological (Fig. 3a, Fig. S2B, D) and functional ERG phenotype (data not shown). In contrast, same-aged sv/sv mutant mice clearly revealed morphological differences: sv/sv mice exhibited a reduced fenestration of the choriocapillaris (CC) [30] and an increasing distance between remaining fenestrae (Fig. 3c), which together with the increased photoreceptor cell death [28] (Fig. S2B, D) may indicate an AMD-relevant phenotype.

**Fig. 4:**
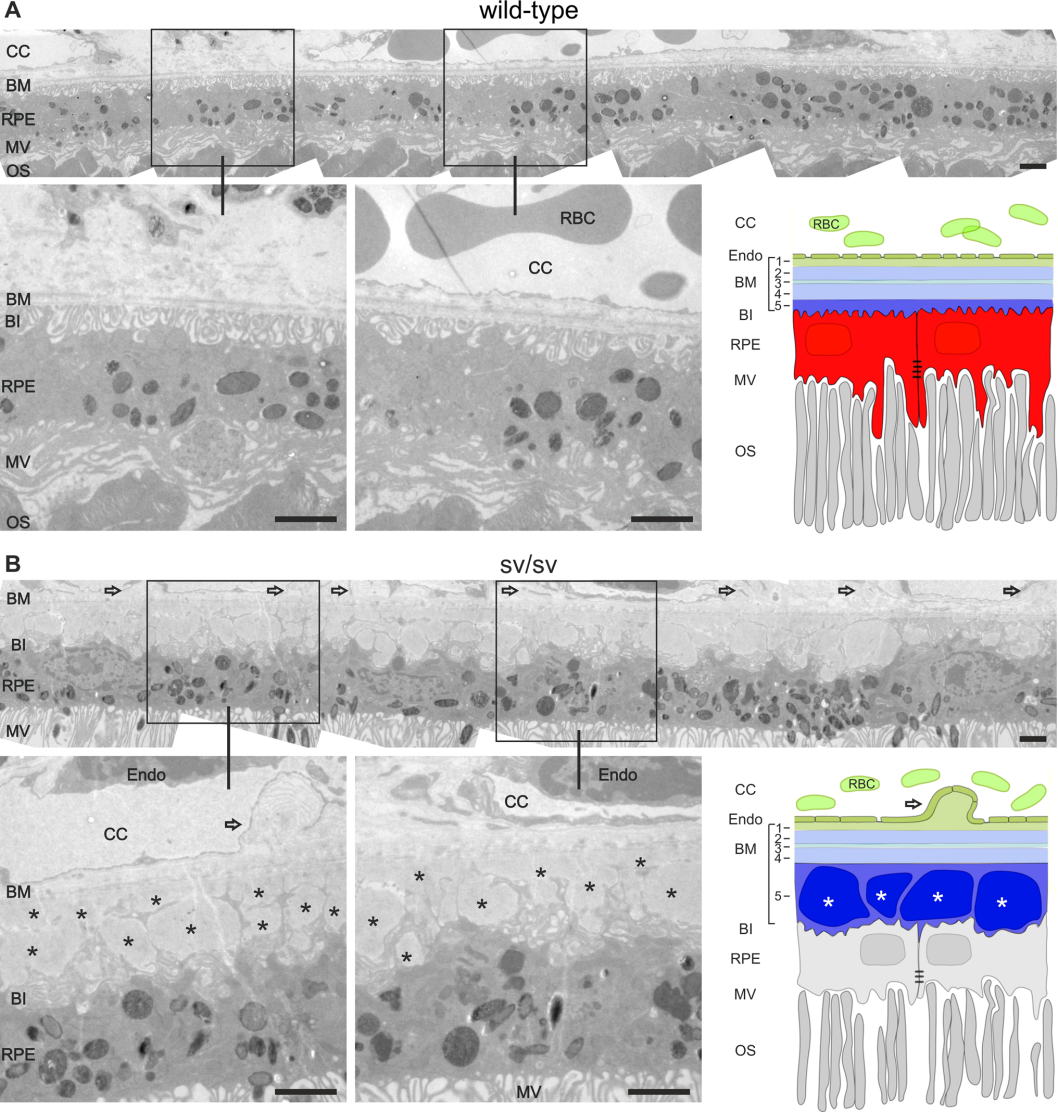
Ultrastructural analyses of the endothelium, the Bruch’s membrane, and the retinal pigment epithelium in wild-type and sv/sv mutant retinae. **a** Outer retina of wild-type mice at the age of 5 months. Note the retinal pigment epithelium (RPE), its association with the Bruch’s membrane (BM), the choriocapillaris (CC), and the photoreceptor outer segments (OS). Note the five layers Bruch’s membrane: *1* basal lamina of the CC; *2* outer collagenous layer, *3* elastic layer, *4* inner collagenous layer, *5* basal lamina of the RPE. **b** Outer retina of same-aged sv/sv mutant mice. *Open arrows* point to protrusions of the choriocapillary endothelium. Basal laminar deposits (*asterisks*) were detected in the thickened basal lamina of the RPE, whereas the basal infoldings (BI) of the RPE cells were distorted. *MV* microvilli, *RBC* red blood cells, *Endo* endothelial cells. *Scale bars* 2 µm

To analyze this feature in more detail, serial sections were collected from 4 wild-type mice, and 9 +/sv and 15 sv/sv mutant mice (5–8-month-old). Typically, as shown for wild-type mice, the apical microvilli of the RPE face the photoreceptor outer segments (OS) (Fig. 4a). Also typically, the basal invaginations of the RPE are firmly attached to the inner layer of Bruch’s membrane, which separates the RPE from the fenestrated endothelium of the CC as shown within different regions of serial sectioned wild-type mice (Fig. 4a). In contrast to this ordered structure, in 10 out of 15 retinae of sv/sv mutant mice (exemplarily shown in Fig. 4b), basal laminar deposits were observed that likely arise from lipoprotein debris [31]. Basal laminar deposits were associated with irregular protrusions of the choriocapillary endothelium (Fig. 4b). Out of 9 +/sv mice between 5 and 8 months, 4 mice developed basal laminar deposits, indicating that heterozygous mice between 5 and 8 months exhibited already an increased risk for altered RPE morphology (data not shown).

### Progression of functional deficits in outer and inner retina function upon myosin VI deletion over age

**Fig. 5:**
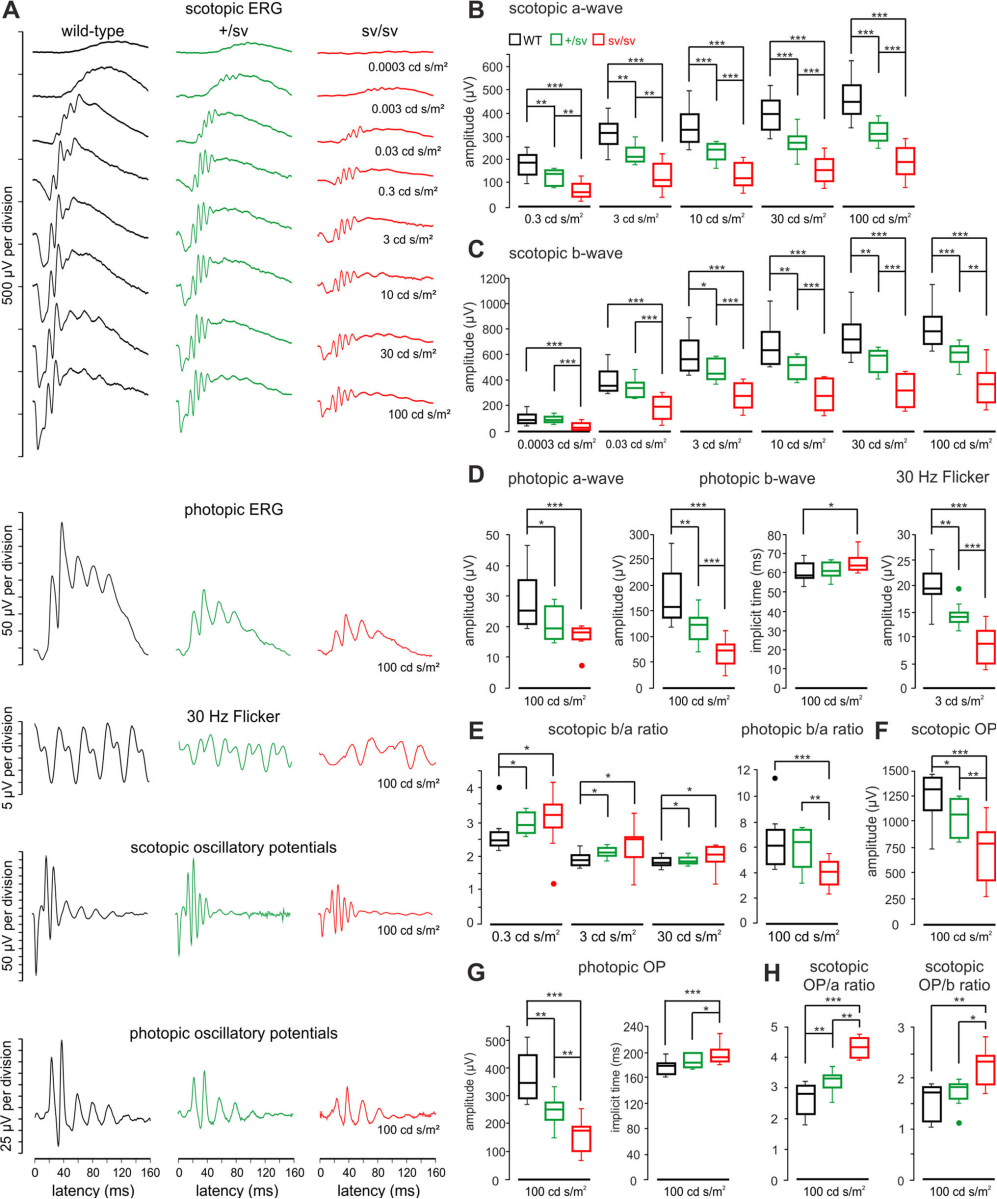
Retinal function of 12-month-old myosin VI mutant mice evaluated by electroretinography. **a** Typical electroretinographic waveforms obtained in wild-type, +/sv and sv/sv mice using the techniques and intensities of the light stimulus as indicated in the figure. **b** Quantification of scotopic a-waves in wild-type (*black*), +/sv (*green*) and sv/sv (*red*) mutant mice revealed a profound reduction of the a-wave amplitude in mutant mice. **c** Quantification of scotopic b-waves revealed a profound reduction of b-wave amplitudes in the mutant mice. **d** Quantification of photopic a-wave amplitude (*left*), b-wave amplitude and implicit time (*middle*) and 30 Hz flicker amplitude (*right*). **e** Scotopic (*left*) and photopic (*right*) b/a ratios with stimulus intensities from 0.3 to 100 cd s/m^2^. **f** Quantification of scotopic oscillatory potentials (OP) revealed a reduction of the OP amplitude in the mutant mice.** g** Quantification of photopic oscillatory potentials (OP) revealed a reduction of the OP amplitude (*left*) and an implicit time increase (*right*) in mutant mice. **h** Scotopic OP/a and OP/b ratios for a stimulus light intensity of 100 cd s/m^2^

In heterozygous +/sv mutants, ERGs are normal at an age of 1 month but showed a slightly, yet not significant reduced a-wave and b-wave amplitudes later at an age of 5–7 months (data not shown). In combination with the first morphological retinal changes observed in 5–7-month-old +/sv mutants, this indicates a slow progression of a functional ocular phenotype in myosin VI mutant mice over age. Therefore, ERG measurements were performed in an experimental series in at least 12-month-old wild-type, and +/sv and sv/sv mutant animals. Different from 5–7-month-old mice, 12-month-old heterozygote (+/sv) mice now have already developed a profound ocular phenotype, indicating a progression of the pathology with age. Scotopic a-wave (Fig. 5a, b) and b-wave amplitudes (Fig. 5a, c) were significantly decreased in homozygous sv/sv mice at light stimulus intensities from 0.3 to 100 cd s/m^2^ and from 0.0003 to 100 cd s/m^2^, respectively. This also occurs, however, with a significant less pronounced phenotype, for the heterozygous gene deletion, and not in +/sv mice for light intensities of 0.03 cd s/m^2^ and lower (Fig. 5c). For photopic ERG measurements, the amplitudes of a-waves and b-waves were also significantly smaller in +/sv mice than in wild-type littermates and comparatively reduced again in sv/sv mutant mice (Fig. 5a, d). No differences were found between implicit times obtained in the three animal groups in scotopic measurements (data not shown), while for photopic b-waves, the implicit time in sv/sv mice was significantly delayed (Fig. 5d), indicating delayed post-photoreceptor responses of cone- but not rod-mediated signals. Amplitudes of ERG responses to photopic 30 Hz flicker stimuli were also significantly reduced in +/sv and sv/sv mutant mice (Fig. 5a, d). 30 Hz flicker latencies were almost the same in wild-type mice and +/sv mutants (33.9 ± 2.7 vs. 33.1 ± 4.3 ms), however, significantly longer in sv/sv mutants (39.3 ± 4.7 ms, *p* = 0.009). Overall, these findings point to both photoreceptor dysfunction and disturbed inner retinal function in myosin VI mutant mice.

To assess how the altered b-wave amplitudes, which originate downstream of the photoreceptors, revealed dependency on photoreceptor function, represented by the a-wave, we analyzed the so-called b/a ratio. This ratio is believed to correlate with the total number of functioning retinal elements [32, 33]. Interestingly, scotopic b/a ratios were increased in both myosin VI mutants, while the photopic b/a ratio was decreased for sv/sv animals, but not for +/sv animals (Fig. 5e). This indicates that the b-wave amplitude is relatively larger than the a-wave for scotopic stimuli in heterozygous and homozygous myosin VI mutant mice, but smaller than the a-wave for photopic stimuli in homozygous myosin VI mutants. Relatively larger and smaller b-wave amplitudes for scotopic or photopic light stimuli, respectively, point to enhanced rod-mediated signals and deteriorated cone-mediated signals on the bipolar cell/amacrine cell level in myosin VI mutant retina.

To validate altered inner retina function deficits, scotopic and photopic oscillatory potentials (OP) were measured as an useful tool to study pathologies related to inner retinal activity, including damage of the optic nerve, retinal ganglion cell loss or its dysfunction or vascular pressure difference during retinal diseases such as glaucoma [34]. Oscillatory potential amplitudes to scotopic (Fig. 5a, f) and photopic (Fig. 5a, g) stimuli were significantly reduced in both myosin VI mutants, with a larger amplitude decrement in sv/sv mice. Similar as for photopic b-wave responses, also the implicit time of photopic OP was significantly delayed (Fig. 5g) confirming disturbed signal transduction at the level of bipolar and/or retinal ganglion cells for cone- but not rod-mediated responses. When OP amplitudes to scotopic stimuli were correlated with the amplitudes of a-waves and b-waves (OP/a and OP/b ratios, respectively), an increased amplitude ratio was obtained for sv/sv mutant mice (Fig. 5h) confirming that scotopic a-waves and b-waves were reduced more than scotopic OP signals in mutant myosin VI mice. In conclusion, our ERG measurements in over 12-month-old myosin VI mutant mice indicate (1) both rod and cone dysfunction in myosin VI mutant mice, (2) reduced inner retinal activity for cone-mediated signals, and (3) enhanced inner retinal activity for rod-mediated signals. Thus, depletion or reduction of myosin VI protein likely has differential effects on cone- and rod-mediated activity in the inner myosin VI mutant retina.

### Myosin VI depletion is correlated with microglia infiltration

**Fig. 6:**
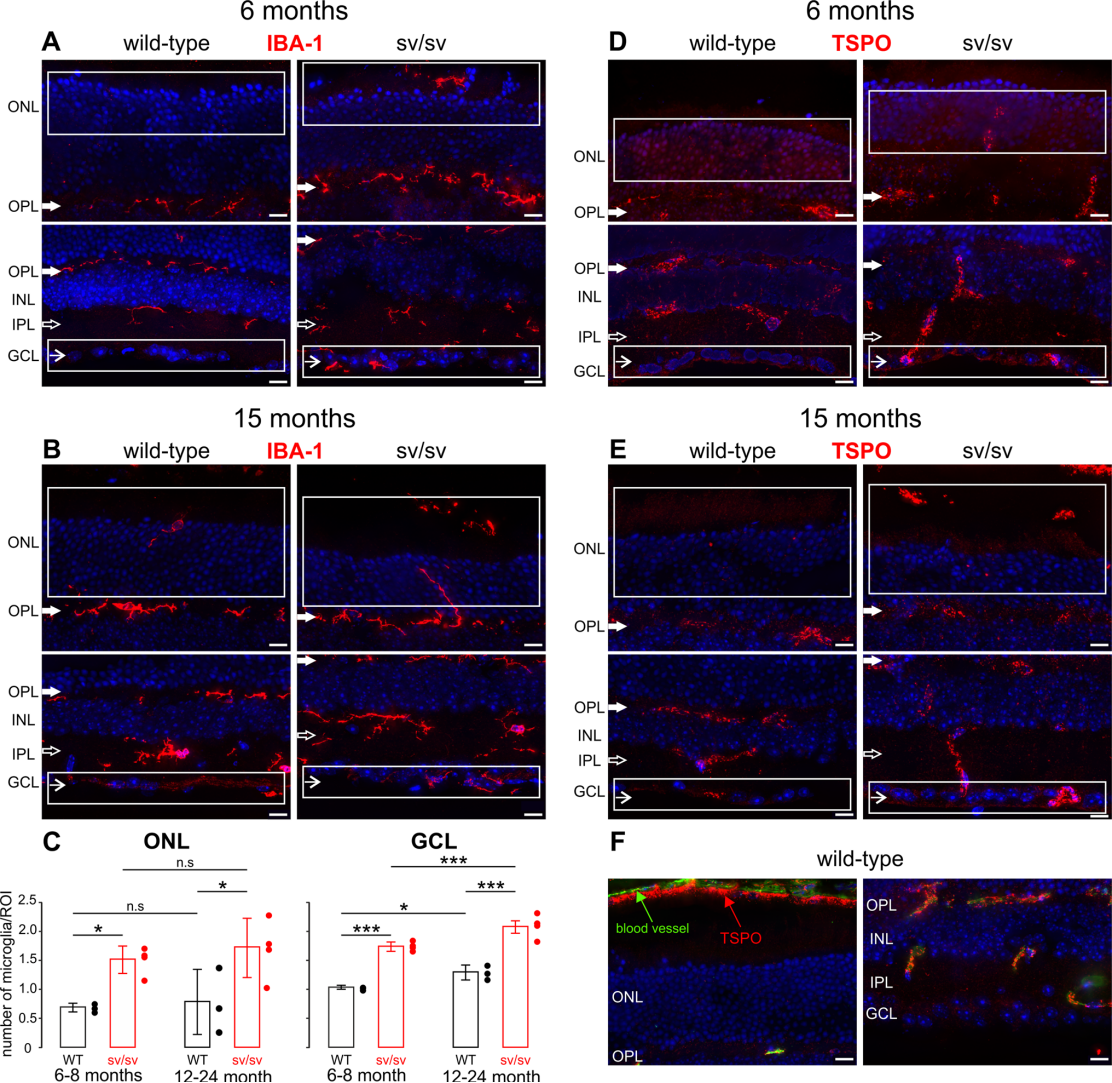
Microglia infiltration in myosin VI mutant retina. Immunostaining of 6- (**a**) and 15-month-old (**b**) retinae of wild-type and sv/sv mutant mice for IBA-1, a marker for microglia. In wild-type retina, IBA-1 immunoreactivity was observed in the outer (OPL *closed arrows*) and inner plexiform layers (IPL *open arrows*). In sv/sv mutant mice, IBA-1-positive microglia staining was additionally noted in the outer nuclear layer (ONL) and also at the level of the ganglion cell layer (GCL *arrows*). *White boxes* indicate region used for IBA-1 quantification shown in **c**. **c** Quantification of IBA-1 labeling in retinae of 6–8- and 12–24-month-old wild-type (*black*) and sv/sv (*red*) mutant mice. 2-way ANOVA with *α* = 0.05; ONL: *p* < 0.01 for genotype, n.s. for age; GCL: *p* < 0.01 for genotype and age. Post-hoc tests performed with Bonferroni adjustment. **d, e** Immunostaining of 6- (**d**) and 15-month-old (**e**) retinae of wild-type and sv/sv mutant mice for TSPO, a marker for reactive retinal microglia. In wild-type mice, TSPO immunoreactivity was found in retinal blood vessel, whereas in sv/sv mutant mice TSPO expression was also found in the GCL and the ONL/OS region. **f** TSPO expression (*red*) in retinal blood vessels (*green* autofluorescence) of adult wild-type mice confirms specificity of the TSPO antibody. *Scale bars* 10 µm

Our results suggest a neurodegenerative phenotype in both the outer and inner retina when expression of myosin VI is precluded or reduced. Retinal degeneration is often accompanied by a migration of microglia into the ONL and the ganglion cell layer (GCL) [12, 13, 35]. Thus, we analyzed the immunoreactivity for the microglial marker IBA-1 in retinal sections of 6–8- (Fig. 6a) and 12–24-month-old (Fig. 6b) wild-type, +/sv (data not shown) and sv/sv mutant mice using high-resolution convoluted fluorescence microscopy. IBA-1 immunoreactivity is typically seen in a ramified pattern in both synaptic layers, the outer plexiform layer (OPL) and inner plexiform layer (IPL), as shown in 6–8-month-old wild-type mice (Fig. 6a). 6–8-month-old +/sv mice, however, did also show an infiltration of IBA-1-positive microglia in the ONL and GCL compared with the wild-type (data not shown). In comparison, at that age, IBA-1 expression was increased in sv/sv mutants (Fig. 6c, 6–8 month). At an age of 12–24 months, sv/sv myosin VI mutant mice exhibited an elevated level of IBA-1 immunoreactivity in the ONL and GCL (Fig. 6c, 12–24 months). In the ONL, no age-dependent differences in IBA-1 immunoreactivity in sv/sv mutant mice were found (Fig. 6c, ONL). In contrast, in the GCL, aged wild-type and sv/sv myosin VI mutant mice showed a significant increase in IBA-1 immunoreactivity (Fig. 6c, GCL).

The translocator protein TSPO has been recently described to be up-regulated in reactive retinal microglia [14, 36]. Therefore, to confirm microglia reactivity, we next analyzed the immunoreactivity for TSPO in retinal sections of 6- and 15-month-old wild-type and sv/sv mutant mice (Fig. 6d, e). In wild-type mice, TSPO immunoreactivity is mainly observed in retinal blood vessels of the OPL, IPL, and the CC (Fig. 6d–f) [36]. In contrast, in sv/sv myosin VI mutant mice, TSPO expression is also observed in the GCL and the ONL/OS region, where also infiltration of IBA-1-positive microglia was observed (compare Fig. 6a, b with Fig. 6d, e).

Taken together, an increased migration of microglia into the GCL and ONL in myosin VI mutant mice may mirror progression of degenerative processes in both the inner and outer retina.

## Discussion

**Fig. 7:**
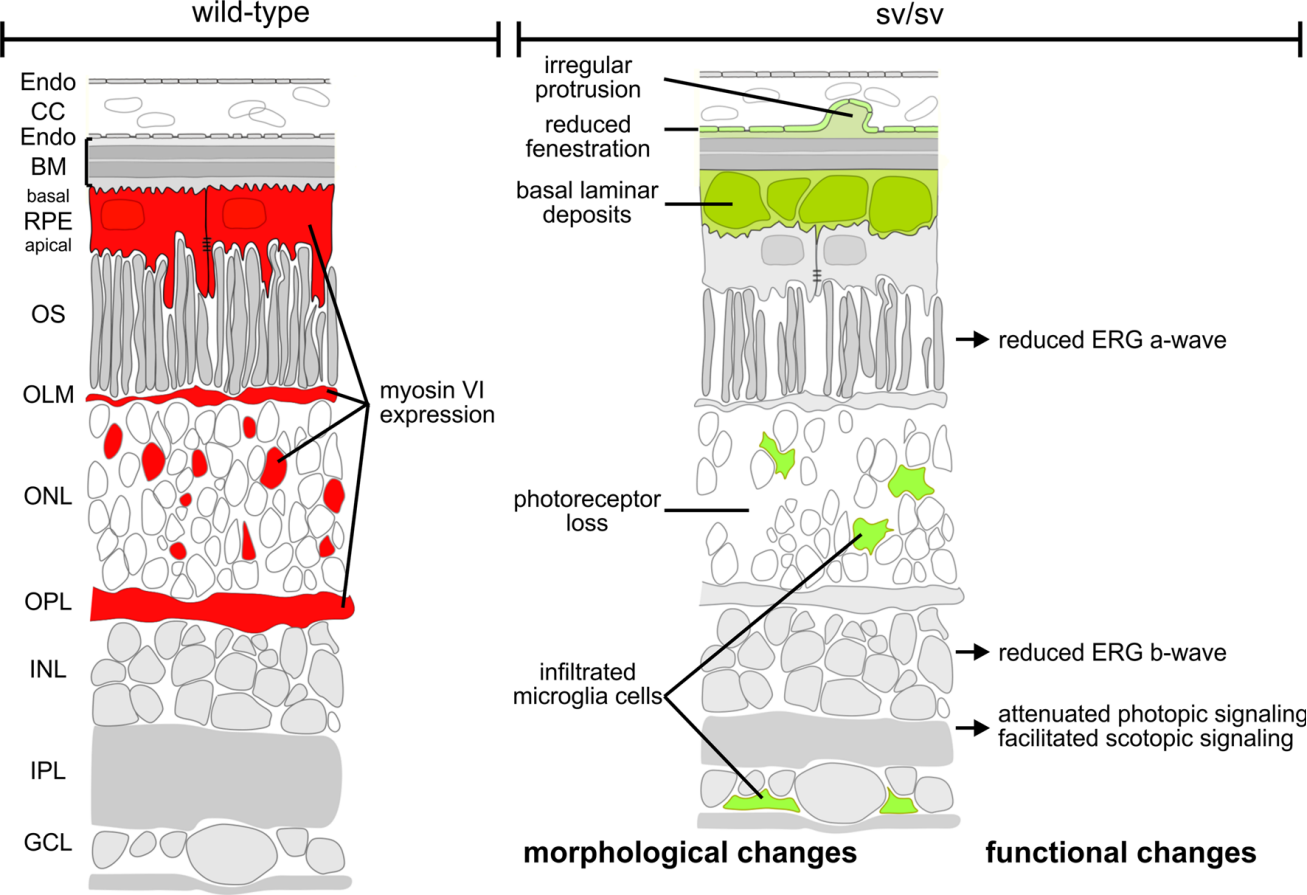
Scheme illustrating morphological and functional alterations in sv/sv mutant retina (*right*) compared with wild-type retina (*left*). Myosin VI immunoreactivity in cells of the RPE, OLM, ONL, and OPL is indicated in *red* in the wild-type retina (*left*) but is lacking in the mutant retina (*right*). Morphological and functional changes in the mutant retina are indicated in *green* (*right*)

In the present study, we showed that the presence of myosin VI is essential for maintaining the structural and functional integrity of the mouse retina (for an overview see Fig. 7). The depletion of myosin VI leads to a visual phenotype that may recapitulate functional and morphological characteristics of glaucoma as well as age-related macular degeneration (AMD) pathology. Myosin VI and its cargo adaptor proteins have previously been suggested to link the entire membrane sorting processes encompassing endocytosis and autophagy [9]. The present study supports a crucial function of myosin VI for linking these processes in the RPE and outer retinal circuits. Our findings suggest that the depletion of myosin VI induces a failure in crosstalk of the protein sorting and organelle clearance pathway(s) and results in sub-RPE basal laminar deposits as well as retina circuit abnormalities.

In the following, we discuss this hypothesis on the basis of our findings in detail. Up to now, the modeling of age-related macular degeneration in mice has been seen critical, as mice lack a macula. Nevertheless, it has been suggested that the mouse may be an useful model system to gain understanding of the pathogenesis of AMD [37, 38]. Only previously, serious concerns of mouse models for ocular disorder-induced mutant phenotypes in general were brought up, due to the finding of a *rd8* mutation in the C57BL/6N mouse strain, which is widely used to produce transgenic mice [20]. Multiple existing mouse models for AMD that have previously thought to meet the critical criteria to gain improved insight in understanding the pathogenesis of AMD therefore may have developed an ocular diseased phenotype unrelated to the gene of interest [20]. Thus, the absence of the *rd8* mutation assured in the present study is a first important prerequisite that allows bringing up a causal relationship with the observed ocular pathologies to myosin VI depletion.

### Myosin IV depletion exhibits characteristics of an AMD-relevant phenotype

Age-related macular degeneration is the most common form of macular degenerations and the leading cause of irreversible blindness in the USA [39]. In contrast to inherited maculopathies, for which mutations in specific genes have been identified, AMD is a multi-factorial disease that is difficult to study because of its late onset in patients (~55 years), complex genetics, and confounding associated environmental risks [40]. Interactions between microglial and RPE cells in the subretinal space are suggested to contribute to altered physiology of RPE cells. Migrated microglial cells are suggested to transform the environment of the retinochoroidal interface into one conductive for the progression and advancement of AMD, including formation of basal laminar deposits between the RPE and Bruch’s membrane [18, 41]. Incidentally, similar basal laminar deposits have also been observed in the *Efemp1* knock-in mouse, an animal that has been highlighted as a model for malattia levantinese and AMD [42].

In the present study, using electron microscopy, basal laminar deposits are found between the RPE and the Bruch’s membrane in most sv/sv and some +/sv mice aged between 5 and 8 months. It is currently assumed that the RPE function to digest retinal outer segment disks is disturbed in AMD, and an alternative lysosomal degradation pathway takes over clearance of damaged organelles, pathogens, and large cytosolic complexes [43]. Over age or following cellular stress, a deterioration of this accumulation of undigested material in lysosomes occurs that modifies their capability to fuse with autophagosomes, with the result of inefficient clearance of damaged components, increased autophagic vacuole accumulation in the cytoplasm and subsequently formation of basal laminar deposits [44]. Taking such a scenario into account, the present finding suggests that myosin VI in RPE is involved in the digestion of photoreceptor outer segment disks. While further studies are essential to strengthen this hypothesis, the basal laminar deposits in myosin VI mutants are possibly the result of a disturbed lysosomal degradation pathway and an incomplete photoreceptor disk membranes clearance.

Additional support for myosin VI being involved in the resorting of membrane during autophagy processes in the RPE comes from the abnormal microglia distribution in the outer retina, which may be a further characteristic feature of AMD [35, 45]. Indeed, morphological studies with retinas of older AMD patients that are already devoid of an intact RPE revealed activated microglia in the photoreceptor layer and subretinal space. In this condition (which is different to our myosin VI-deficient mouse where the RPE is still present), subretinal microglia incorporated rhodopsin-positive particles, indicating phagocytosis of dead photoreceptors [35]. Independent from the presence of a RPE, microglia cells translocate to the subretinal space during aging in the murine and human retina [46], where they are suggested to evoke directly low-grade progressive degeneration [12, 13] and secretion of angiogenic factors [47]. Also, the increased rate in cell death in the ONL in homozygous myosin VI mutant mice confirms photoreceptor degradation. However, compared to other hereditary retinal degeneration models [26], the cell death rate in myosin VI-deficient mice is rather low, which may correspond to the very protracted degeneration process in human AMD.

As shown in the Results section, a functional decline is found in the mutant mice, manifesting itself by a reduction of all amplitudes, both scotopic and photopic. In contrast, changes in implicit times were observed only in cone-driven post-receptoral responses, i.e., implicit times of photopic OP, photopic b-wave, and photopic 30 Hz flicker. There are several ocular conditions that lead to a delay of cone-related implicit time, such as hereditary photoreceptor degeneration [48], birdshot retinochoroidopathy [49], central areolar choroidal dystrophy [50], disturbance in ocular blood pressure [51], the Laurence-Moon-Bardet-Biedl syndrome [52], diabetic retinopathy [53] and congenital stationary night blindness [54], to name a few. Moreover, the decrease in photopic 30 Hz flicker amplitudes and delays in implicit time has been considered a predictor of progressed neovascularization in patients with central vein occlusion [55]. It remains to be elucidated which pathology leads to the changes of electroretinographic parameters we observed in our study.

Interestingly, Dab2, a myosin VI adaptor protein, has been shown to be expressed in the retina, where it is discussed to be involved in VEGF receptor endocytosis and angiogenesis in the retina [56]. Future studies may thus reinvestigate the AMD-related phenotype described here through disturbed flicker responses, microglia infiltration and basal laminar deposits in the context of possible Dab2 dysfunctions.

### Myosin IV mutant mice exhibit characteristics similar to a glaucoma phenotype

Glaucoma is considered a multi-factorial disease [57], and many of the proposed mechanisms, are traditionally linked to elevated intraocular pressure (IOP)-related factors. In addition, the activation of microglia may facilitate disease progression and retinal ganglion cell loss independent of IOP elevation. While AMD and glaucoma typically occur in separate patient groups [58], both diseases may present a co-morbidity [15].

We found that myosin VI is expressed in the OPL that is a dense network of synapses between horizontal cells, bipolar cells and photoreceptors. Localization of myosin VI close to calbindin, a marker for horizontal cells, and in part with PKCα that labels rod bipolar cell dendrites in the outer retina [23] suggests a role of myosin VI adaptor proteins for cargo and protein resorting during the synaptic transmission process in the outer retina. The role of myosin VI may include maintenance of vesicle transport back and forth to the endoplasmatic reticulum within the photoreceptor and/or horizontal cell synapses [9]. The partial co-localization of myosin VI in the OPL with AP-2 that couples myosin VI to clathrin-mediated endocytosis [8, 25] confirms synaptic localization in photoreceptor and/or horizontal cell synapses. Only recently, a role of myosin VI in inner hair cell synapses for coupling of endocytosis and exocytosis as well as for maintenance of surface membrane has been shown [7, 8]. The disturbance of outer retinal synaptic activity due to the myosin VI deficiency may result in altered inner retinal activity: Oscillatory potentials mirror inner retinal function, including inhibitory circuits between amacrine cells and bipolar cells/ganglion cells [33, 59, 60]. Thus, the decreased photopic b/a ratio and the reduced oscillatory potentials for photopic stimuli in aged heterozygous and homozygous myosin VI mutant mice have to be regarded as disturbed inner retinal circuits to cone-evoked signals, subsequent to failure of proper myosin VI-based cargo of vesicles in cone photoreceptors and/or horizontal cells. In line with our observation in the myosin VI mutant retina, outer and inner retinal circuits alter their function after photoreceptor degeneration and show oscillatory activity patterns in the *rd1* retina [61, 62].

Oscillatory potential components may also correlate with retinal arteriolar caliber in patients with diabetes [63], suggesting that there is a connection between retinal dysfunction and microvasculature changes. Within this context, the elevated b/a ratio and increased OP/a and OP/b ratios in response to scotopic stimuli need special consideration. In an earlier study, Kergoat and Lovasik [64] provided evidence that the rod system is more vulnerable than the cone system to transient alterations of the retinal vascular perfusion pressure (RVPP). When the susceptibility of scotopic OP to altered retinal perfusion was analyzed in volunteers, a defined component of the OP did exhibit pronounced gain in amplitude when retinal vascular pressure was increased [64]. It thus may be interesting in future to investigate scotopic response amplitudes of OP in myosin VI mutant mice in the context of increased vascular pressure. The significant increase of IBA-1-labeled microglial cells at the level of the ganglion cells observed in myosin VI mutants hints at pathological/degenerative processes in the inner retina. In line with that, activated microglia cells have been identified in glaucoma models [65–67], where they may point to stress signals sent from neurons to the glial cells involved in low-grade progressive degeneration and optic neuropathy [12, 13, 18].

Among the various adaptor proteins that interact with myosin VI during the process of autophagosome maturation is optineurin [68]. Sequence variants in the gene for optineurin have been reported to be associated with normal tension glaucoma, a subtype of primary open-angle glaucoma [69, 70]. Expression studies showed that optineurin is not only found in nerve fibers and retinal ganglion cells, as expected for a glaucoma-candidate gene, but also in the RPE. A possible differential spatio-temporal distribution of the myosin VI-bound adaptor proteins in either IPL or OPL may thus couple the entire cycle of sorting of membranes back to the plasma membrane and delivery to degradation processes [9].

Taken together, the present study not only introduces myosin VI as a gene potentially responsible for genetic predispositions of either AMD or glaucoma. The finding, moreover, untangles the endocytotic sorting process per se as a potential cause of these disastrous diseases, as this process is highly sensitive to metabolic demand, mitochondrial damage, age or stress, and could explain the multi-factorial disease of AMD and glaucoma. Not only myosin VI but also cargo adaptor proteins such as Dab2, GIPC, Tom1, optineurin, NDP52, or T6BP [9] may be considered in the future as other candidate genes involved in hereditary AMD forms or genetic predispositions. The human gene *MYO6*, located on chromosome 6q13, maps with the deafness locus DFNA22 and DFNB37 [71–73]. A single case of a family member with DFNB37 that suffered from retinitis pigmentosa [71] is reported. Whether an ocular phenotype occurs over age in either DFNB37 or DFNA22 families is currently unknown, as the currently clinical observed members are still young [71].

### Electronic supplementary material

Below is the link to the electronic supplementary material.

**Fig. S1** Genotyping of myosin VI mutant mice for the *rd8* mutation. Samples from sv/sv and +/sv mutants and wild-type controls were probed for the *rd8* mutation by PCR and compared to a positive control samples obtained from Ccl2/Cx3cr1 double knockout (CCDKO) animals (17). In *rd8* mutants, pre-digestion with *Nde*I yielded characteristic 45 and 199 bp fragments found in CCDKO-positive controls but not in myosin VI mutants and control littermates (TIFF 853 kb)

Photoreceptor cell death in sv/sv mutant retinae. (**A**) 1.5-month-old sv/sv mutant retina displays no/minor differences of cell death rate compared to +/sv retina. (**B**) In contrast, 5−6-month-old sv/sv mutant retina shows an increase in cell death rate in the ONL. Quantitative plots showing cell death rate at 1.5 (**C**) and 5-6 months (**D**) in wild-type, +/sv and sv/sv. *Scale bars*: 50 µm (TIFF 3160 kb)

## Abbreviations

AMD: Age-related macular degeneration
AP-2: Adaptor protein 2
BI: Basal invaginations
BM: Bruch’s membrane
CC: Choriocapillaris
CCDKO: Ccl2/Cx3cr1 double knockout mouse line
Endo: Endothelial cells
ERG: Electroretinogram
GCL: Ganglion cell layer
INL: Inner nuclear layer
IOP: Intraocular pressure
IPL: Inner plexiform layer
MV: Microvilli
OLM: Outer limiting membrane
ONL: Outer nuclear layer
OP: Oscillatory potentials
OPL: Outer plexiform layer
OS: Outer segment
PKCα: Protein kinase C α
RBC: Red blood cells
ROI: Region of interest
RPE: Retinal pigment epithelium
+/sv: Heterozygous Snell’s Waltzer myosin VI-deficient mouse line
sv/sv: Homozygous Snell’s Waltzer myosin VI-deficient mouse line
TEM: Transmission electron microscopy
TUNEL: Terminal deoxynucleotidyltransferase (TdT) dUTP nick end labeling
VGLUT1: Vesicular glutamate transporter 1
