# Supplementary figures and images for: Deletion of myosin VI causes slow retinal optic neuropathy and age-related macular degeneration (AMD)-relevant retinal phenotype
Source: Cell Mol Life Sci. 2015 May 6;72(20):3953–69. doi: 10.1007/s00018-015-1913-3 (PMC4575690; doi:10.1007/s00018-015-1913-3)

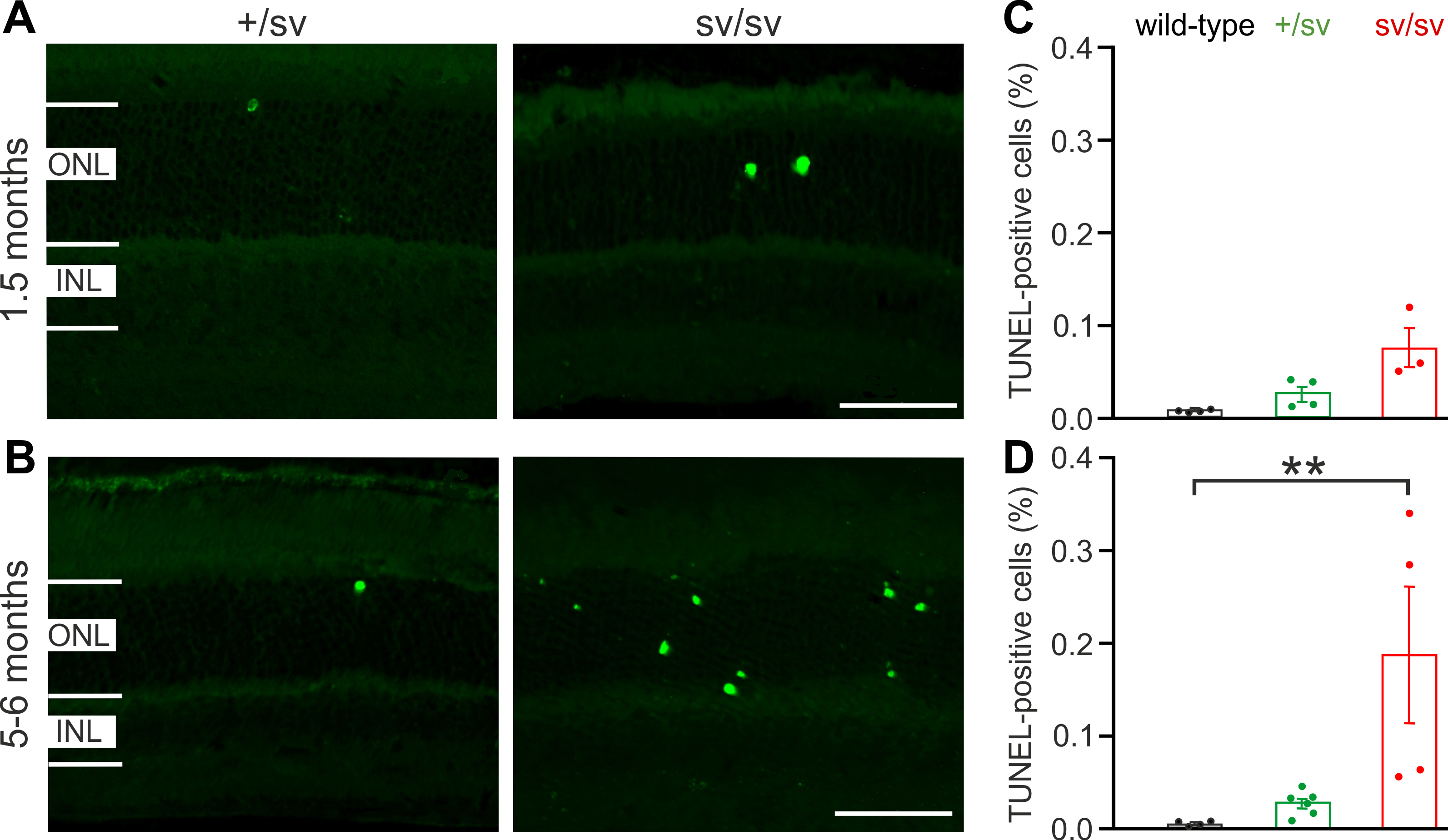

Supplement: Supplementary file 2 — Photoreceptor cell death in sv/sv mutant retinae. (A) 1.5-month-old sv/sv mutant retina displays no/minor differences of cell death rate compared to +/sv retina. (B) In contrast, 5−6-month-old sv/sv mutant retina shows an increase in cell death rate in the ONL. Quantitative plots showing cell death rate at 1.5 (C) and 5-6 months (D) in wild-type, +/sv and sv/sv. Scale bars: 50 µm (TIFF 3160 kb) [file 18_2015_1913_MOESM2_ESM.tif]
